# Total Enteral Nutrition Facilitates Wound Healing Through Preventing Intestinal Atrophy, Keeping Protein Anabolism and Suppressing Inflammation

**DOI:** 10.4021/gr2009.08.1307

**Published:** 2009-07-20

**Authors:** Yutaka Suzuki, Naruo Kawasaki, Mitsuyoshi Urashima, Hironori Odaira, Takuji Noro

**Affiliations:** a Department of Surgery, International University of Health and Welfare; b Division of Clinical Research & Development, Jikei University School of Medicine

**Keywords:** Total enteral nutrition, Total parenteral nutrition, Wound healing, Rats, Cytokine

## Abstract

**Background:**

In clinical settings, early total enteral nutrition (TEN) is known to reduce the postoperative complication and infection rate as well as duration of postoperative stay compared with total parenteral nutrition (TPN) in a variety of critical conditions. We aimed to compare effects TEN and TPN on wound healing and explore its possible mechanisms using rat model.

**Methods:**

Seven days after operation for inserting enteral tube into gastric space for TEN, Sprague-Dawley rats were made burn (15 mm) in the back. Rats were administrated with either TEN (N = 17) or TPN (N = 15) and evaluated condition of wound healing as well as serum/urine immunological and biochemical parameters at 28 days.

**Results:**

Burned area was significantly reduced in TEN than in TPN group. Although body weight, serum levels of total protein, albumin and transferrin were the same levels between the two groups, urine nitrogen and intestinal atrophy were significant in TPN group. Conversely, weight of small bowel showed positive linear relationship with levels of parameters calculated as follows: [medication nitrogen quantity – (urine nitrogen + feces nitrogen)]/[medication nitrogen – feces nitrogen quantity]. Weights of spleen and tumor necrotizing factor-a levels in serum were higher in TPN than in TEN.

**Conclusions:**

These results suggest that TEN may facilitate wound healing compared with TPN through preventing intestinal atrophy, keeping protein anabolism and suppressing inflammation.

## Introduction

In clinical settings, early total enteral nutrition (TEN) is known to reduce the postoperative complication and infection rate as well as duration of postoperative stay compared with total parenteral nutrition (TPN) in a variety of critical conditions: postoperative recovery [1-3], abdominal trauma [4, 5], pancreatitis [6], burn [7-9]. To support the superiority of TEN to TPN obtained as clinical evidences, rodent’s models demonstrated a couple of mechanisms that TEN reduced cytokine production after operation or burn [10, 11], TPN increased apoptosis in the small bowel mucosa [12] and bacterial and endotoxin translocation [13-15]. However, these evidence using rodents models were fragmented and not comprehensive. Therefore, we aimed to compare effects of TEN and TPN on wound healing by measuring a variety of parameters using burned rat model.

## Materials and Methods

### Treatment of animals

All studies were carried out in compliance with the institutional guidelines of animal experiments at Jikei University School of Medicine. Male Sprague-Dawley rats at 11 weeks (Charles River Japan Inc., Yokohama, Japan) were fed with CRF-1: standard meal for rats (Oriental Co., Tokyo, Japan). After making sure rats’ condition healthy by spending 7 days, either intragastric root for TEN or intravenous root for TPN was obtained under anesthesia induced by administrating 40 mg/kg of pentobarbital intraperitoneally. For TEN group, a polyvinyl catheter (5 Fr) (Japan Sharwood, Tokyo, Japan) was inserted into intragastric space of the rat and the other end of catheter was guided subcutaneously to the back, and then connected with joint pipe with stainless steel by opened abdominal operation. For TPN group, opened abdominal operation alone was performed.

One week after abdominal operation, burns were made on the center of rats’ back under anesthesia by intraperitoneally administrating pentobarbital. Briefly, after shaving the back, 42 rats had burn of 15 mm in diameter by pressing an electrical soldering iron at 200 °C for 30 seconds. Two days after making burn, debridement was performed for skin of wound tissue under anesthesia with pentobarbital. Three days after making burn, absorbent cotton covered with OpSite Wound (Smith and Nephew Inc., Florida, USA) was put on wound tissue to absorb exudative solution. Wound healing was leave opened between 7 and 28 days after making burn.

At the day of making burn (defined as day 0), either TEN or TPN was started. For TEN, the other end of joint pipe was connected to a swivel via fixing spring with polyethylene tube through harness on the back of rat. For TPN, a small skin incision was made in the inguinal region of the rat to insert a silicon catheter (inner diameter: 0.5 mm, outer diameter: 1.0 mm; Kaneka Medix, Tokyo, Japan) into the right femoral vein to reach vena cava inferior. The other end of the catheter was guided subcutaneously to the posterior aspect of the neck, and then connected to a swivel via harness on the back of rat. The skin wound for insertion of the catheter was closed by a stitch of 3-0 silk. Rats of TEN group had incision at inguinal region alone.

Either TEN or TPN, nutrition was continuously infused through polyethylene tube connecting to the other side of swivel for 24 hours per day using perista pump (Watoson-Marlow 502S; Nikkisou Co. Ltd., Tokyo, Japan) without peroral feeding. Rats belonging to either TEN or TPN group were administrated nutrition 60 kcal/day from day 0 to day 1 and 80 kcal/day from day 2 to day 28. To confirm normal range of parameters, control rats (N = 12) not treated with burn or operation were fed with the same calorie during the same period of time. Components of nutrients used for TEN: Twinline^®^ (Otsuka Pharmaceutical Co., Ltd.; Tokyo, Japan), and for TPN: Unicaliq^®^ (N) (Termo Co., Tokyo, Japan) + Multamin^®^ (Sankyo Co. Ltd., Tokyo, Japan) + Mineralin^®^ (Takeda Pharmaceutical Co. Ltd., Tokyo, Japan) + Intrafat^®^ (20%) (Takeda Pharmaceutical Co. Ltd., Tokyo, Japan) + 20%(W/W) Choline chloride (Wako Pure Chemical Ind., Osaka, Japan), solutions were shown in Table 1. These solutions were made up in clean bench. As a control group, rats without operation nor treatment for burn were fed CRF-1 for the same calorie during the same experimental period to know normal ranges of serum/urine parameters for nutrition and inflammation.

**Table 1:** Components of nutrients used for TEN or TPN (/100 kcal)

Component		TEN^*1^	TPN^*2^
Carbohydrate		14.68 g	20.59 g
Lipid	LCT	0.812 g	0.374 g
	MCT	1.968 g	0 g
Amino acid		4.05 g	3.53 g
Total nitrate		0.6 g	0.55 g
Volume		100 ml	120 ml
Calorie/N		176	182
Calorie of non protein		83.7 kcal	85.9 kcal
Calorie of non protein/N		140	157

*1: Twinline^®^ (Otsuka co. ltd, Tokyo, Japan)

*2: Unicaliq^®^ (N) + Multamin^®^+ Mineralin^®^ + Intrafat^®^ (20%) + 20% Chorine chloride

At final day of observation, opened abdominal operation was performed under anesthesia with intraperitoneally administrated pentobarbital for blood sampling from portal vein and vena cava inferior. After sacrificed with total bleeding, spleen was resected for weight measure.

### Outcome measures

Degree of wound healing was estimated by area of wound at day 0 of making burn and day 28 of sacrificed using Image analysis software (IPAP-WIN: Sumika Technoservice Co., Osaka, Japan) for measuring area. Body weights were measured at day 0 and day 28.

Urine and stool were collected during day 23, day 24 and day 25. Urine was frozen and stool was freeze-dried and packaged until nitrogen measures. Urine and feces nitrogen quantity was determined daily by a chemiluminescence technique from day 0 to day 28 after burn. Nitrogen accounts = medication nitrogen – (urine nitrogen + feces nitrogen); Prices = nitrogen accounts/(medication nitrogen – feces nitrogen)

Serum protein levels were measured as following combinations: prolyl hydroxylase by enzyme immunoassay (EIA)-kit (Fujiyakuhin Co., Ltd. Saitama, Japan); total protein and albumin by A/G B-test WAKO (Wako Pure Chemical Ind., Osaka, Japan); transferrin by EIA-kit (Panafirm Laboratories Co. Ltd., Kumamoto, Japan); sialic acid by sialic acid measurement kit (Kyokuto Pharmaceutical Industrial Co., Ltd. Tokyo, Japan); endotoxin assay kit by toxi-color (Seikagaku Co., Tokyo, Japan); tumor necrotizing factor (TNF)-a, interleukin (IL)-6, IL-4 and IL-10 by ELISA kit (Biosource International Inc., California, USA); IL-8 by Rat GRO/CINC ELISA system (Amersham Lifescience Inc., Buckinghamshire, UK). Biochemical parameters were measured with an automatic serum analyzer (Model 7150, Hitachi Ltd., Tokyo, Japan).

### Statistics

Significant differences among TEN, TPN and control group were estimated with Kruskal-Wallis test and defined as significant when p-value was less than 0.05. On the other hand, significant differences between two groups: TEN and TPN; TEN and control; TPN and control; were estimated with Mann-Whitney (Two-sample Wilcoxon rank-sum) test. The difference was defined significant when p-value was less than 0.016 according to Bonferroni correction. All statistical analyses were performed using STATA version 8.0 (Stata Corporation, College Station, TX, USA).

## Results

### Changes of body weight and nutrition markers

**Figure 1:**
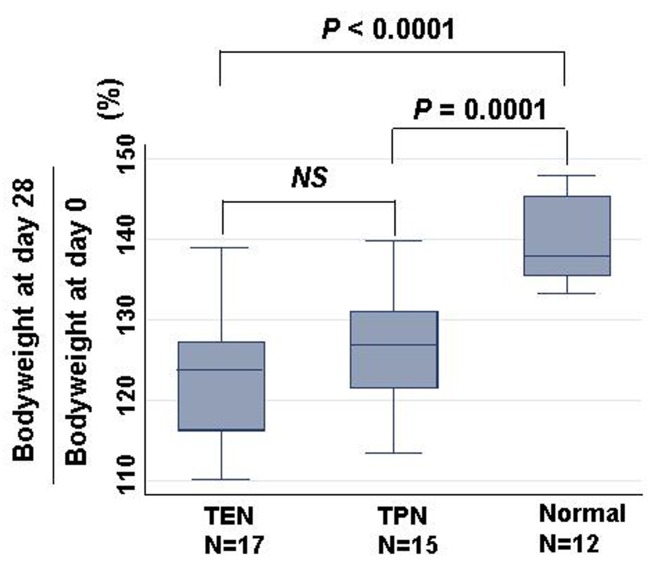
Comparison of bodyweight changes among TEN, TPN, and normal groups. Changes were calculated as bodyweight at day 28/ bodyweight at day 0 (%). Statistical difference was evaluated with Man Whitney U test.

Total calorie intake was not different among TEN (N = 17; 2223.0 ± 10.0 kcal), TPN (N = 15; 2246.1 ± 11.0 kcal) and control groups (N = 12; 2203.7 ± 12.0 kcal). Percent changes of body weight at day 28 divided by bodyweight at day 0 were compared among TEN, TPN, and normal groups (Fig. 1). Increase of body weight was equivalent between TEN (N = 17; 122.4 ± 7.8%) and TPN (N = 15; 126.3 ± 7.2%). While body weights of burned rats treated with either TEN or TPN were significantly less than control rats without burn (N = 12; 139.8 ± 5.1%).

Nutritional markers were compared among three groups (Table 2). Although medication nitrogen quantity and feces nitrogen was least in TPN group, urine nitrogen was most. Thus, the prices were least in TPN, second in TEN, and most in control group. Serum levels of total protein, albumin and transferrin at day 28 were equivalent between TEN and TPN, whereas those of control group were significantly higher than TEN and TPN.

**Table 2:** Effects of TEN on nutrition

Biomarker	TEN N = 17	TPN^*2^ N = 15	Control^*3^ N = 12	Kruskal-Wallis ^*1^
Medication nitrogen quantity	947.8 ± 27.3	881.2 ± 15.1^‡^	1515.7 ± 8.1^‡^	0.0001
Urine inside nitrogen quantity	659.2 ± 63.6	755.1 ± 73.2^†^	625.4 ± 42.1^‡^	0.0077
Feces inside nitrogen quantity	62.2 ± 30.7	22.9 ± 8.6^†^	365.4 ± 7.1^‡^	< 0.0001
Nitrogen accounts	226.3 ± 87.2	103.3 ± 80.7^†^	524.8 ± 34.9^‡^	< 0.0001
Prices	25.3 ± 9.1	11.9 ± 9.3^†^	45.6 ± 3.3^‡^	< 0.0001
Total protein (g/dl)	5.4 ± 0.3	5.3 ± 0.4	4.9 ± 0.2^‡^	0.0004
Albumin (g/dl)	1.8 ± 0.1	1.8 ± 0.1	2.2 ± 0.1^‡^	< 0.0001
Transferrin (mg/ml)	3.19 ± 0.41	3.13 ± 0.62	3.16 ± 0.50^‡^	0.0005

*1: Statistical differences were calculated based on Kruskal-Wallis equality of populations rank test. Statistical significance was defined when p-value was less than 0.05.

*2: Value of TPN was compared with TEN by correcting with Bonferroni.

*3: Value of control was compared with TEN.: † p < 0.016, ‡: p < 0.005.

### Effects of TEN on wound healing

**Figure 2:**
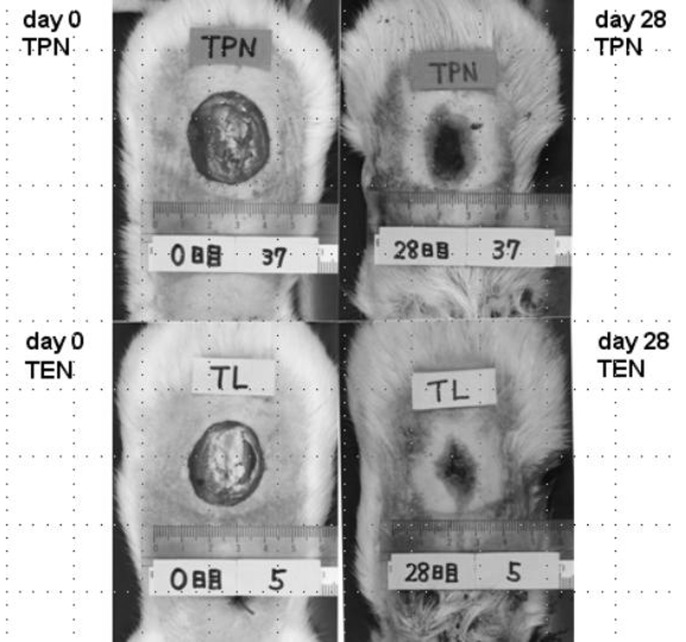
Difference of burned area at day 0 (left panel) and day 28 (right panel) between TPN (upper) and TEN (lower).

**Figure 3:**
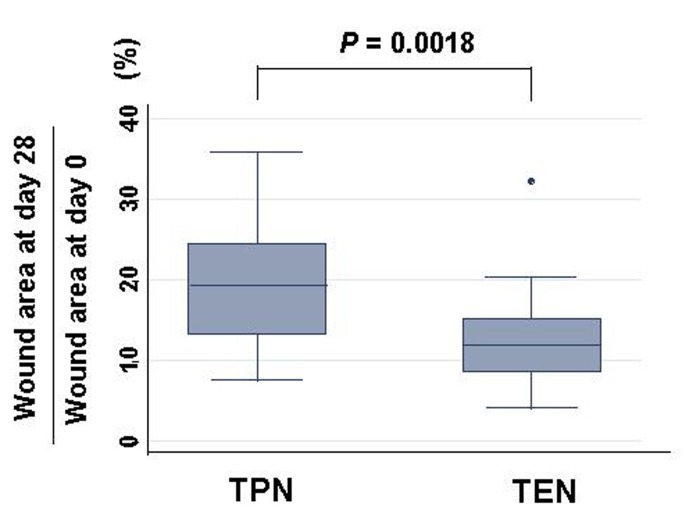
Changes of burned area: burned area at day 28 divided by wound area at day 0 (%) in either TPN or TEN. Statistical difference was evaluated with Man Whitney U test.

Typical wound areas of rats’ back at day 28 treated with either TEN or TPN were shown as Figure 2. Burned area was significantly smaller in rats treated with TEN (12.7 ± 1.6% of burned area at day 0) than with TPN (19.0 ± 2.3% of burned area at day 0) (Fig. 3). Serum levels of prolyl hydroxylase were significantly lower in TEN (449.4 ± 98.2 ng/ml) than TPN group (953.2 ± 611.3 ng/ml) (*P* = 0.0004), which were more than control (286.5 ± 41.3 ng/ml) (*P* = 0.0001).

### Effects of TEN on morphology of small intestine

**Figure 4:**
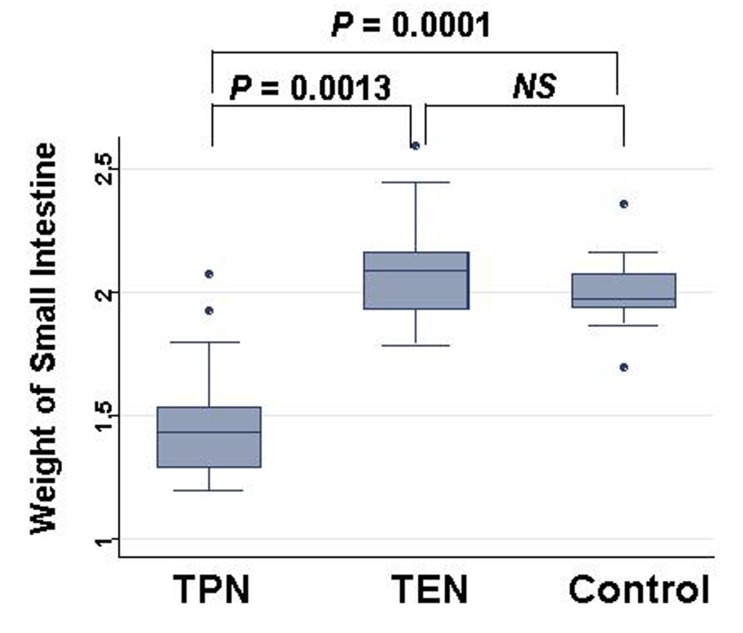
Comparison of weights of small intestine at day 28 among TPN, EN and control group. Statistical difference was evaluated with Man Whitney U test.

**Figure 5:**
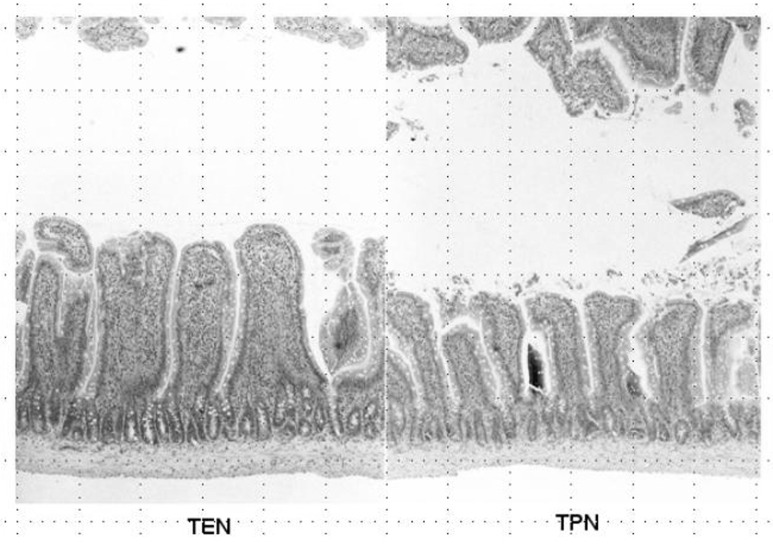
Hematoxylin and eosin staining of jejunum obtained from TEN (left panel) and TPN (right panel).

**Figure 6:**
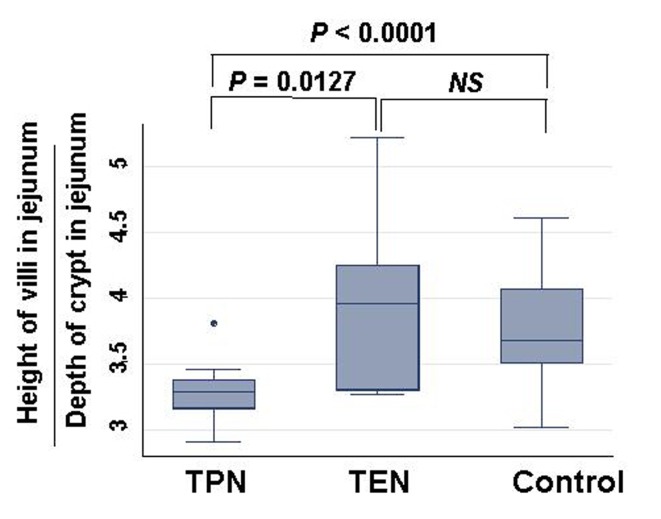
Comparison of ratio: length of villi divided by depth of crypt in jejunum among TPN, EN and control group. Statistical difference was evaluated with Man Whitney U test.

**Figure 7:**
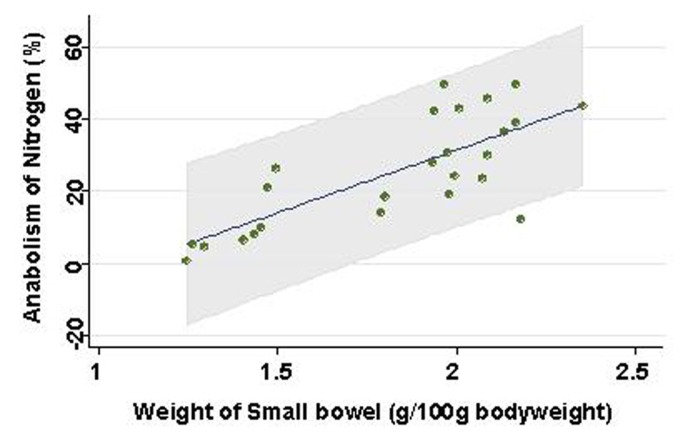
Association between weight of small bowel and prices. Line and gray area are showing linear regression and 95% confidence interval, respectively.

Weight of small intestinal loop per 100 g of body weight was heavier in TEN (2.08 ± 0.22 g) and control group (2.00 ± 0.16 g) than in TPN group (1.48 ± 0.26 g), although no significant difference existed between TEN and control group (Fig. 4). Then, length of villi and depth of crypt were measured in duodenum, jejunum and ileum under hematoxylin eosin staining among TEN, TPN, and control group: Examples of hematoxylin and eosin staining of jejunum were shown (Fig. 5). Then, ratio: length of villi divided by depth of crypt in jejunum was compared among TPN, TEN and control group (Fig. 6). The ratio was significantly smaller in TPN group than in TEN (P = 0.012). Weight of small bowel per 100 g of bodyweight showed positive and linear association with anabolism of nitrogen (Fig. 7).

### Effects of TEN on immunological and biochemical markers

Other parameters possibly related with wound healing were also compared among TEN, TPN, and control group (Table 3). Among cytokines measured in this experiment, only TNF-a (*P* = 0.0042) were significantly higher in TPN group than in TEN. Weights of spleen were also heavier in TPN than in TEN (*P* < 0.0001) or control group (*P* < 0.0001).

**Table 3:** Effects of TEN on inflammation

Types	Biomarker	TEN N = 17	TPN N = 15	Control N = 12	Kruskal-Wallis ^*1^
Immune	Sialic acid (mg/dl)	109.6 ± 12.2	125.5 ± 21.5	75.6 ± 6.8^‡^	< 0.0001
	IL-4 (pg/ml)	5.4 ± 8.3	10.6 ± 12.7	9.7 ± 10.6	NS
	IL-6 (pg/ml)	2.4 ± 6.7	56.7 ± 91.6	0 ± 0	0.0061
	IL-8 (pg/ml)	193.2 ± 144.0	283.5 ± 277.0	122.8 ± 46.4	NS
	IL-10 (pg/ml)	3.6 ± 9.8	18.2 ± 42.4	3.9 ± 13.4	NS
	TNF-α (pg/ml)	1.7 ± 4.2	3.9 ± 4.1^‡^	1.3 ± 1.7	0.0087
	Endotoxin (pg/ml)	18.5 ± 6.2	20.1 ± 6.1	5.7 ± 1.5	0.0012
	Spleen (g/100g bodyweight)	0.25 ± 0.03	0.71 ± 0.40^‡^	0.22 ± 0.03	< 0.0001
Liver	AST (U/l)	129.0 ± 42.1	567.5 ± 1005.2^*2^	327.0 ± 698.6	NS
	ALT (U/l)	36.4 ± 9.4	92.1 ± 145.6	30.7 ± 7.5	NS
	Alkaliphosphatase (U/l)	360.5 ± 74.4	569.1 ± 222.8^‡^	491 ± 109.4	0.0012
	LAP (IU/l)	54.6 ± 1.5	70.1 ± 25.5	71.6 ± 6.1^†^	0.0066
	Direct bilirubin (mg/dl)	0.113 ± 0.057	0.196 ± 0.117^‡^	0.088 ± 0.016	0.0018
	Indirect bilirubin (mg/dl)	0.015 ± 0.014	0.055 ± 0.075^†^	0.030 ± 0.012	NS
Renal	BUN (mg/dl)	18.5 ± 2.3	22.7 ± 4.5^‡^	16.4 ± 15.5	< 0.0001
	Cr (mg/dl)	0.46 ± 0.05	0.55 ± 0.08^‡^	0.42 ± 0.05	< 0.0001
Metabolism	Glucose (mg/dl)	157.3 ± 5.5	116.8 ± 32.7^‡^	141.9 ± 17.5	0.0004
	Triglyceride (mg/dl)	51.8 ± 19.6	23.1 ± 11.1^‡^	35.8 ± 17.3^†^	0.0002
	Total cholesterol (mg/dl)	60.8 ± 11.5	55.5 ± 10.4	54.0 ± 10.8	NS

*1: Statistical differences were calculated based on Kruskal-Wallis equality of populations rank test. Statistical significance was defined when p-value was less than 0.05. *2. Two high levels of AST (2864, 2994) were included in TPN group. †: p < 0.016, ‡: p < 0.005.

Both serum levels of direct (*P* = 0.0025), indirect bilirubin (*P* = 0.0041) and alkaliphosphatase (*P* = 0.0083) were significantly higher in TPN group than in TEN group. Moreover, BUN (*P* = 0.0058) and creatinine (*P* = 0.0021) were also higher in TPN group than in TEN group. In contrast, plasma glucose (*P* = 0.0015) and triglyceride levels (*P* = 0.0001) were lower in TPN group than in TEN group.

## Discussion

In this study, wound healing was faster in TEN group than in TPN group, in spite of equivalent body weight changes after burn. There were few original articles to demonstrate superiority of TEN to TPN in wound healing using rat model [16-18]. Judging from data of nitrogen accounts and prices in this experiment, TEN can direct more anabolic state than TPN, although total protein, albumin, transferrin were equivalent between TPN and TEN group. In addition, blood urea nitrogen (BUN) that is one of protein metabolites was lower in TEN than in TPN in our study. TPN treated malnourished rats gained more weight with greater body fat formation than TEN group but had lower nitrogen [19]. Thus, our results and a previous report suggest that TEN may facilitate wound healing by maintaining protein anabolism more than TPN.

Weight of small intestinal loop was heavier in TEN and control group than in TPN group, although no significant difference existed between TEN and control group. Moreover, ratio length of villi divided by depth of crypt was significantly smaller in TPN group than in TEN and control group, which may be consistent with previous reports that morphometry revealed an increased submucosal thickness while intestinal circumference markedly decreased in TPN-treated rats compared with TEN [20, 21]. TPN may keep gut little stress, conversely cause mucosal atrophy. Furthermore, these morphological changes induced by TPN were demonstrated to associate with reduced lymphocytes, increased gut permeability and enhanced bacterial translocation [22-26], which can increase risk of postoperative sepsis and postoperative morbidity/mortality [13, 27]. Small intestinal atrophy was shown to affect nitrogen metabolism to a greater extent than liver by-pass [28], which was also reconfirmed in our study that weight of small bowel showed positive linear relationship with levels of nitrate anabolism.

Among cytokine production, serum levels of TNF-a were significantly lower in TEN than in TPN group. TPN increases the expression of TNF-a mRNA in organ tissues and systemic TNF-a production, and reduces the survival rate of rats after thermal injury, but TEN does not [11]. Thus, differences in cytokine levels between TEN and TPN in our study were consistent with previous studies. Moreover, increased weight of spleen confirmed in this study was not pointed out previously to our knowledge. Decreased stimulation to intestinal immunity may be compensated by hypertrophy of spleen at least in part.

Serum levels of bilirubin and alkaliphosphatase were significantly higher in TPN group than in TEN group. During TPN, hepatic concentration of the important intracellular antioxidant glutathione was reported to decrease [29]. Capacity of hepatic drug metabolism was shown to decrease in rats treated with TPN [30, 31]. Moreover, BUN and creatinine were also higher in TPN group than in TEN group. Rats given TEN after ischemic acute renal failure have improved renal function compared with rats given TPN [32]. These suggest that TEN may protect multiple organs against failure in critical conditions. In contrast, plasma glucose and triglyceride levels were lower in TPN group than in TEN group, of which meanings remain unknown.

In conclusion, TEN may facilitate wound healing compared with TPN through preventing intestinal atrophy, keeping protein anabolism and suppressing inflammation.
